# Hepatic primary neuroendocrine carcinoma: about a new case

**DOI:** 10.11604/pamj.2015.20.254.5710

**Published:** 2015-03-17

**Authors:** Hasna Derouich, Fouad Haddad, Mohamed Moukhlissi, Wafaa Hliwa, Ahmed Bellabah, Wafaa Badre

**Affiliations:** 1 Gastroenterology, Oncology Departemnt Ibn Rochd Hospital, Casablanca, Morocco

**Keywords:** Carcinoma, neuroendocrine, liver

## Abstract

We report a new case of Primary hepatic neuroendocrine carcinoma admitted in our hospital and revealed in 53 years man by epigastric pain and flush syndrome. A liver biopsy with immunohistochemical study confirmed the original location of a neuroendocrine carcinoma. After 12 cures of Chemotherapy and a follow up of 12 months, the patient is still in complete remission.

## Introduction

Endocrine tumors are characterized by a common phenotype general markers (i.e., neuron-specific enolase, chromogranin, synaptophysin) and hormonal secretion products. Primitive hepatic localization is very rare, representing less than 0.3% of all endocrine tumors of the digestive system with a female predominance; the sex ratio is 1.6 / 1, with an average age of 51 [1, 2].

## Patient and observation

**Figure 1:**
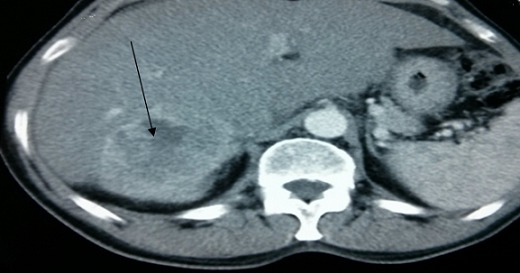
CT scan image of a PHNEC patient; CT scan showing a liver mass (segment VI and VII)

We report a case of primary hepatic neuroendocrine carcinoma (PHNEC) admitted in our departement. A 53 years old patient, known diabetic, admitted for epigastric pain combined with diarrhea, abdominal distension and flush syndrome. This evolved in a deterioration of general condition. The clinical exam attested a facial reddening, hepatomegaly. An abdominal ultrasound showed a giant hepatic hemangioma of the posterior segment. A thoraco-abdominal-pelvic CT objective tumor lesion localized segment VI and VII of the liver with ascites (Figure 1).

Digestive endoscopy was performed for the patient and no Neoplasm was found in the stomach, duodenum, colon, or rectum. Capsule endoscopy for the small intestine was normal. Serum 5-HT, chromogranin A (CgA), and urinary 5-hydroxyindoleacetic acid (5-HIAA) examinations were very high with values of 225 ng/ml and 19 mg/24h successively. CT scan of the thorax was normal. The patient were Alpha Fetoprotein (AFP-), Carcinoembryonic antigen (CEA -) and Carcinoma Antigen (CA19-9-).

**Figure 2:**
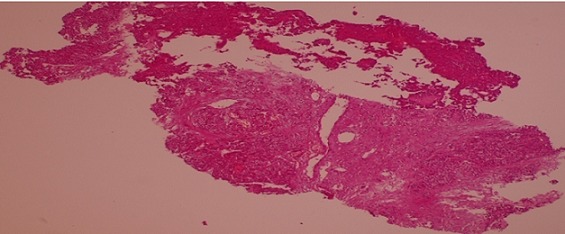
Microscopic image at low magnification showing tumor proliferation

**Figure 3:**
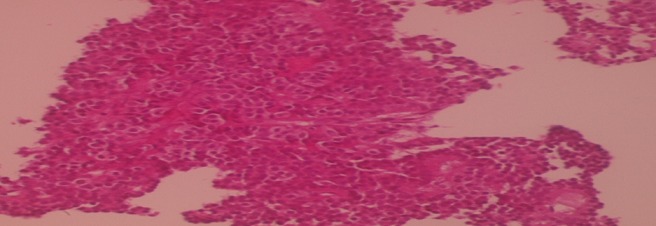
Hematoxylin eosin staining

**Figure 4:**
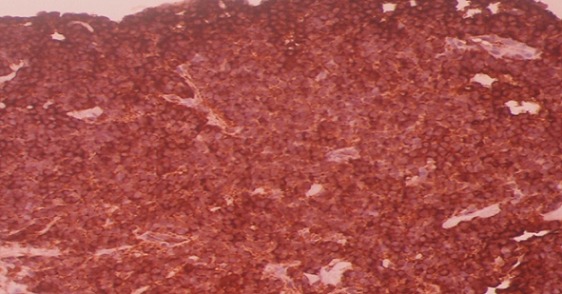
Immunohistochemistry, chromogranin A

A liver biopsy with immunohistochemical study confirmed the diagnosis of primary neuroendocrine carcinoma (Figure 2, Figure 3, Figure 4). The patient has received chemotherapy with Cisplatin 100 mg/m2/day J1 and Etoposide 100 mg/m2/day J1 to J3 for 6 months, with good clinical and radiological improvement, and then he undergone 6 months more treatments: 12 months of treatments in total. After 18 months of follow up, the patient is in complete remission and we opted for a clinical and radiological monitoring.

## Discussion

Primary hepatic neuroendocrine carcinoma is rare [1, 2], clincal symptoms are not specific, and imaging is fundamental to guide diagnosis. Serum 5-HT or hydroxyindoleacetic acid (5-HIAA) 24 h urine levels may be effective markers with sensitivity of 73% and a specificity of more than 90%. Serum (CGA) is a sensitive marker in the diagnosis with a sensitivity of 87-100% and a specificity of 92%, but diagnosis confirmation is the histoligical examination after biopsy or surgery [3].

It is difficult to differentiate PHNEC from other solid tumors, especially hepatocellular carcinoma or hepatic metastasis of other tumor; therefore, postoperative pathologic examination is the main method for a final diagnosis [1, 4]. Treatment includes several methods, surgery, systemic chemotherapy and transcatheter arterial chemoembo-lization [5, 6]. Prognosis depends on the initial stage of the disease. Research indicated that the recurrence rate at 5 years was 18% and the survival rate at 5 years was 74% -78% [7].

## Conclusion

Primary hepatic neuroendocrine carcinoma remains a rare diagnosis, requiring careful biological, endoscopic, radiological and histological examinations to eliminate the secondary localization of extrahepatic tumor.
